# Virtual reality relaxation for hospitals: A novel stress-reduction intervention for patients, families, and staff

**DOI:** 10.1016/j.fhj.2025.100234

**Published:** 2025-03-08

**Authors:** Simon Riches, Grace Williams

**Affiliations:** a King's College London, Department of Psychology, Institute of Psychiatry, Psychology & Neuroscience, London, SE5 8AF, United Kingdom; b King's College London, Social, Genetic & Developmental Psychiatry Centre, Institute of Psychiatry, Psychology & Neuroscience, London, SE5 8AF, United Kingdom; c South London and Maudsley NHS Foundation Trust, BR3 3BX, United Kingdom

**Keywords:** VR, wellbeing, healthcare, digital mental health, healthtech

## Abstract

Hospitals can be stressful environments for patients, families and staff. There are many wellbeing interventions to manage stress, but they can be difficult to implement in busy clinical environments. Novel solutions are needed to improve the hospital experience. Virtual reality (VR) interventions for relaxation, wellbeing and stress reduction involve users interacting with calming environments using a head-mounted display. Numerous studies in the general population and psychiatric settings suggest that VR relaxation is effective. Given these stress reduction outcomes within psychiatric hospitals, there is huge potential for general hospitals to implement VR. VR could be offered to patients who may be experiencing significant distress or perioperative anxiety, as well as to staff and families in waiting rooms. This is an exciting opportunity for patients, researchers, and clinicians with experience in VR therapies to guide general hospitals and make recommendations for clinical applications so that they can experience the benefits of VR relaxation.

General hospitals can be highly stressful environments for patients, families and staff. For patients and families, challenges include the experience of being personally unwell or caring for a loved one, in addition to treatment delays, cancellations, inadequate staffing and strikes. For clinical staff, short-staffing, high staff turnover and huge workloads and clinical demand for treatments in the context of limited resources have led to poor staff wellbeing and high levels of stress and burnout. There are many wellbeing interventions to manage stress, but they can be difficult to implement and carry out in busy clinical environments, and so novel solutions are needed to improve the hospital experience.

Healthcare is shifting to ever-greater use of technology and digital interventions. One highly promising innovation is virtual reality (VR) interventions for relaxation, wellbeing and stress reduction, which involve users interacting with calming environments using VR headsets and 3D, immersive audiovisual content.^1^ VR relaxation is an engaging and popular innovation, and is effective at reducing short-term stress and anxiety, even from brief use in single sessions. Numerous psychological studies in the general population and clinical settings suggest that VR relaxation is effective, either as a self-help intervention or when facilitated by a clinician or therapist.^1^^,^^2^ Pilot studies indicate that VR relaxation is feasible and helpful to both patients and staff when it has been introduced in psychiatric hospitals, including for significantly mentally unwell and distressed psychotic patients on acute psychiatric wards.^3^ A study of VR relaxation within an acute psychiatric ward found post-VR significant increases in relaxation, happiness, connectedness to nature, and decreases in stress, anxiety and sadness.^4^ Another study found that VR relaxation can be offered as a helpful alternative when a physical ‘calm room’ is not available in psychiatric wards and can improve wellbeing for patients.^5^ Research has found similar wellbeing benefits for staff in psychiatric settings.^6^

Given these stress reduction outcomes within psychiatric hospitals, there is scope for general hospitals to benefit in similar ways, particularly as many patients, families and staff may already be using VR for general purposes, such as for gaming and entertainment at home. In clinical settings, a review of VR for adolescents in general hospitals found that VR relaxation can improve pain and anxiety, especially due to immersive elements of VR-based interventions.^7^ A study on a paediatric ward indicated that VR relaxation is feasible and acceptable for patient participants and clinicians offering the intervention, and can reduce anxiety, pain and stress, and increase happiness.^8^ Another study found that as little as 10 minutes of VR relaxation can be effective at reducing perceived stress and anxiety for staff working in intensive care units.^9^ Given that the psychiatric literature indicates that people who are unwell and in distress can engage with VR relaxation, there is potential for this novel intervention to be offered more broadly to physically unwell patients, including people in intensive care units and acute medical wards who may also be experiencing significant distress. VR relaxation can be implemented as a distraction tool to manage perioperative anxiety, discomfort and pain. Other implementation strategies might include offering VR relaxation as a staff support intervention or to families in waiting rooms and other settings; VR relaxation may be beneficial when individuals cannot engage with other relaxation techniques, such as mindfulness and meditation.

Potential limitations and barriers to implementing VR in general hospitals include issues of cost where there are very limited and stretched resources, which may limit scalability. For successful implementation, hospitals would need to invest in VR therapies, including equipment and training staff and patients to use VR. Furthermore, hospitals would need to allocate physical space for the VR therapies to occur. This could be integrated into other wellbeing interventions that are already offered in many hospitals, such as wellbeing hubs, that may be in separate rooms away from wards. However, the portable nature of VR headsets may help to overcome limited room availability, as VR interventions can be offered to patients at their bedside, or even during procedures.^7^^,^^8^ Other potential barriers may include general user experience and cybersickness, but this is much improved with modern headsets and the risk is minimal.^10^ Resistance to change and working with technologies may also be a barrier to implementation.^11^ Hospital staff, patients and carers, and other stakeholders would need to learn about these new digital interventions in order to engage with them, this would require advocates for novel VR therapies to support delivery within hospitals. This could involve VR facilitators or clinicians with VR experience demonstrating the equipment, for example, in waiting rooms.

Current research into VR therapies for general hospitals is limited in terms of methodological rigour, and future research requires robust studies to support this promising area of clinical care. There is a need for randomised controlled trials that use standardised measures of psychological constructs, such as patient anxiety, staff wellbeing, and family or carer stress.^7^^,^^8^^,^^12^ Future research could involve quantitative and qualitative studies of VR in general hospitals to evaluate this intervention, including the appeal across all patient age groups and other demographic categories. Future research could also explore the extent to which people could engage with VR in general hospitals as a self-help intervention, or whether it would need a clinician or therapist to facilitate it, which may be dependent on the needs of the individual or group being offered VR.

Based on existing research, VR relaxation could be offered more widely to patients, carers and staff. This is an exciting opportunity for patients, researchers and clinicians with experience in VR-based therapies to guide general hospitals and make recommendations for clinical applications.^11^ This will enable patients and staff working within these organisations to experience the benefits of VR relaxation.

## CRediT authorship contribution statement

**Simon Riches:** Writing – review & editing, Writing – original draft, Methodology, Investigation, Conceptualization. **Grace Williams:** Writing – review & editing, Writing – original draft, Methodology, Investigation.

## Declaration of competing interests

The authors declare that they have no known competing financial interests or personal relationships that could have appeared to influence the work reported in this paper.
